# Emotional and Behavioral Profiles in Neurodevelopmental and Neuromuscular Disorders: A Comparative Study Using the Child Behavior Checklist

**DOI:** 10.3390/children13080996

**Published:** 2026-07-27

**Authors:** Daniela Pia Rosaria Chieffo, Federica Moriconi, Valentina Delle Donne, Valentina Arcangeli, Valentina Massaroni, Angelica Marfoli, Luca Liberati, Giulia Settimi, Brenno Martelli, Sofia Vannuccini, Chiara Veredice, Gabriele Sani, Eugenio Maria Mercuri

**Affiliations:** 1 Clinical Psychology Unit, Fondazione Policlinico Universitario Agostino Gemelli IRCCS, 00168 Rome, Italy; 2 Complex Operational Unit of Child Neuropsychiatry, Fondazione Policlinico Universitario Agostino Gemelli IRCCS, 00168 Rome, Italy; 3 Department of Women, Children and Public Health, Università Cattolica del Sacro Cuore, 00168 Rome, Italy; 4 Department of Health Science and Public Health, Faculty of Medicine and Surgery, Università Cattolica del Sacro Cuore, 00168 Rome, Italy; 5 Complex Operational Unit of Clinical and Emergency Psychiatry, Fondazione Policlinico Universitario Agostino Gemelli IRCCS, 00168 Rome, Italy; 6 Department of Neuroscience, Section of Psychiatry, Università Cattolica del Sacro Cuore, 00168 Rome, Italy

**Keywords:** Autism Spectrum Disorder, Attention-Deficit/Hyperactivity Disorder, Duchenne Muscular Dystrophy, Child Behavior Checklist

## Abstract

**Highlights:**

**What are the main findings?**

- Children with ADHD showed the highest overall emotional–behavioral burden, whereas ASD and SLD were characterized by greater internalizing difficulties and DMD by a distinct emotional–behavioral profile.
- Using the same standardized CBCL/6–18 assessment enabled direct comparison of emotional and behavioral functioning across neurodevelopmental and neuromuscular conditions.

**What is the implication of the main finding?**

- A transdiagnostic assessment framework may facilitate the identification of both shared and disorder-specific emotional–behavioral patterns across heterogeneous pediatric populations.
- Integrating standardized behavioral screening with individualized clinical assessment may support more targeted psychosocial care and multidisciplinary decision-making.

**Abstract:**

**Background**: Neurodevelopmental disorders, including Autism Spectrum Disorder (ASD), Attention-Deficit/Hyperactivity Disorder (ADHD), and Specific Learning Disorder (SLD), as well as neuromuscular conditions such as Duchenne Muscular Dystrophy (DMD), are frequently associated with emotional and behavioral difficulties that may affect children’s functioning and family well-being. However, direct comparisons across these heterogeneous clinical populations remain limited. This study aimed to compare emotional and behavioral profiles across children and adolescents with ASD, ADHD, SLD, and DMD using the Child Behavior Checklist (CBCL/6–18). **Methods**: This cross-sectional comparative study included 197 children and adolescents (ASD: 47; SLD: 50; ADHD: 50; DMD: 50) assessed at the Clinical Psychology Unit of the Fondazione Policlinico Universitario Agostino Gemelli IRCCS (Rome, Italy) between 2017 and 2024. Emotional and behavioral functioning was evaluated using the parent-report CBCL/6–18. Group differences were examined using one-way ANOVA or Welch’s ANOVA, as appropriate, followed by Tukey’s HSD or Games–Howell post hoc comparisons. **Results**: Significant group differences emerged for Internalizing Problems, Externalizing Problems, and Total Problems (all *p* < 0.001). ADHD showed the highest overall emotional and behavioral burden, with 76% of participants scoring in the clinical range for both Internalizing and Total Problems and 60% for Externalizing Problems. Elevated internalizing difficulties characterized ASD and SLD, whereas children with DMD showed generally lower CBCL scores, although 26% fell within the clinical range for Internalizing Problems. Effect sizes were moderate to large (η^2^ = 0.168–0.273), supporting the presence of both shared and disorder-specific emotional and behavioral patterns across heterogeneous developmental conditions. **Conclusions**: The findings highlight the heterogeneity of emotional–behavioral profiles across neurodevelopmental and neuromuscular conditions. The use of a common standardized assessment framework enabled the identification of both shared and disorder-specific patterns, supporting the potential value of a transdiagnostic approach to individualized assessment. Future longitudinal and multi-informant studies are warranted to clarify developmental trajectories and improve the identification of clinically meaningful emotional and behavioral needs across heterogeneous pediatric populations.

## 1. Introduction

Neurodevelopmental disorders are a heterogeneous group of conditions characterized by atypical brain development that affects cognitive, behavioral, emotional, and adaptive functioning throughout childhood. According to the *Diagnostic and Statistical Manual of Mental Disorders*, *Fifth Edition* (DSM-5), Autism Spectrum Disorder (ASD), Attention-Deficit/Hyperactivity Disorder (ADHD), and Specific Learning Disorder (SLD) are among the most common pediatric neurodevelopmental disorders [1]. ASD is characterized by persistent deficits in social communication and interaction together with restricted and repetitive patterns of behavior and interests [1,2]. ADHD is characterized by developmentally inappropriate levels of inattention and/or hyperactivity-impulsivity [1,3]. SLD involves persistent difficulties in reading, writing, and/or mathematics despite adequate intellectual functioning and appropriate educational opportunities [1].

Although these disorders differ in their core clinical manifestations, they are all associated with emotional and behavioral difficulties that may substantially affect children’s overall functioning and represent an important component of their clinical presentation. Understanding these emotional and behavioral manifestations is therefore essential for a comprehensive characterization of children’s clinical functioning beyond their primary diagnostic features.

The Child Behavior Checklist (CBCL/6–18) is a widely used parent-report instrument for assessing these domains in children and adolescents aged 6–18 years. It provides standardized measures of internalizing and externalizing problems, as well as an overall profile of emotional and behavioral functioning [4,5]. Although the CBCL is widely validated, its sensitivity may vary across different clinical populations, particularly in conditions such as ASD and neuromuscular disorders where symptom presentation may differ [6,7]. Nevertheless, its standardized structure makes it particularly suitable for comparing emotional and behavioral profiles across heterogeneous clinical populations.

A review of the literature highlights the extensive use of the CBCL/6–18 for identifying emotional and behavioral problems in children and adolescents with various psychopathological conditions and neurodevelopmental disorders, including ASD [8,9], SLD [10,11,12], and ADHD [13,14,15,16].

Previous studies have consistently reported elevated levels of emotional and behavioral difficulties in individuals with ASD. The CBCL/6–18 findings indicate elevated scores on the Withdrawn/Depressed, Social Problems, Thinking Problems, and Attention Problems scales, together with frequent overlap between internalizing and externalizing symptoms [6,8,9].

ADHD is primarily associated with externalizing behaviors. Difficulties in self-regulation, frustration tolerance, and emotional functioning are also common and are frequently accompanied by anxiety or depressive symptoms [13,14,17].

Individuals with SLD are more likely to develop both internalizing and externalizing problems than their typically developing peers. Studies using the CBCL/6–18 have consistently reported attention problems, social difficulties, and a predominance of internalizing symptoms, particularly anxiety and depression, in this population [10,11,12,18].

Despite the growing literature on individual neurodevelopmental disorders, direct comparisons of emotional and behavioral profiles across ASD, ADHD, and SLD remain limited.

Available comparative studies nevertheless suggest that emotional and behavioral difficulties differ across these conditions, although the pattern and severity of symptoms vary according to the underlying diagnosis [15]. Operto et al. [15] reported emotional and behavioral problems across all groups, with individuals with ASD exhibiting greater difficulties in socialization, mood, and somatic complaints than those with ADHD and SLD.

Despite its widespread use, the CBCL/6–18 has some limitations. Parent reports may underestimate internalizing problems, particularly in adolescents, and the findings may be influenced by within-group variability, including age, cognitive functioning, and comorbidities [7,19,20,21].

Within this framework, extending behavioral investigations to pediatric conditions beyond neurodevelopmental disorders may provide additional insights into shared and disorder-specific emotional and behavioral characteristics.

Although Duchenne Muscular Dystrophy (DMD) is primarily classified as a neuromuscular disorder rather than a neurodevelopmental condition, increasing evidence indicates that affected children frequently experience emotional and behavioral difficulties that extend beyond the consequences of physical disability. Examining DMD alongside neurodevelopmental disorders within the same standardized behavioral framework may therefore help distinguish shared and disorder-specific emotional and behavioral profiles, thereby providing a broader understanding of psychological functioning across heterogeneous pediatric clinical populations.

DMD is a severe X-linked recessive disorder characterized by progressive muscular degeneration due to mutations in the dystrophin gene. While primarily recognized as a neuromuscular condition, DMD has profound implications for emotional and behavioral functioning, given the psychological and social challenges associated with progressive physical limitations and shortened life expectancy [22].

Children and adolescents with DMD frequently experience internalizing problems such as anxiety and depression, stemming from physical restrictions, social isolation, and the chronic stress of managing a life-limiting condition [23]. Hinton et al. [24] reported that social behavior problems are prevalent in boys with DMD, with 34% showing clinically significant ratings on the CBCL/6–18 Social Problems scale. This suggests the potential involvement of the central nervous system beyond the disease’s direct physical manifestations.

Although less frequently reported, externalizing behaviors may also emerge as responses to frustration and increasing dependence on caregivers. The CBCL/6–18 has been employed in studies of DMD to evaluate these profiles, revealing increased scores in anxiety, withdrawal, social problems, and, less consistently, externalizing behaviors compared with typically developing peers [25,26,27]. Furthermore, these emotional and behavioral difficulties are often compounded by the psychosocial burden experienced by families, emphasizing the need for comprehensive assessments and interventions.

Despite the growing evidence describing emotional and behavioral functioning within individual diagnostic groups, direct comparisons across ASD, ADHD, SLD, and DMD remain scarce. In particular, behavioral research on DMD has largely developed independently from studies of neurodevelopmental disorders, limiting the identification of shared and disorder-specific emotional and behavioral patterns across these heterogeneous pediatric populations.

Using a common standardized measure such as the CBCL/6–18 may help address this gap by enabling direct comparisons across clinically distinct conditions, thereby facilitating the identification of both shared and disorder-specific emotional and behavioral patterns.

Based on these considerations, the present study aimed to compare emotional and behavioral functioning across children and adolescents with ASD, ADHD, SLD, and DMD using the CBCL/6–18. Specifically, the study sought to: (1) compare CBCL/6–18 Internalizing, Externalizing, and Total Problems across the four diagnostic groups; (2) examine whether distinct emotional and behavioral profiles characterize each diagnostic group using a common standardized assessment framework; and (3) identify both shared and disorder-specific emotional and behavioral patterns across neurodevelopmental and neuromuscular conditions. By providing a direct comparison of these clinically distinct populations using the same standardized behavioral measure, this study aims to advance understanding of emotional and behavioral functioning across heterogeneous pediatric clinical populations.

## 2. Materials and Methods

### 2.1. Participants

This cross-sectional comparative study analyzed a dataset including a total of 197 children and adolescents with ASD (*n* = 47), SLD (*n* = 50), ADHD (*n* = 50), and DMD (*n* = 50) who attended the Clinical Psychology Unit and the Child Neuropsychiatry Unit of Fondazione Policlinico Universitario Agostino Gemelli IRCCS, Rome, and who underwent cognitive, emotional, or learning assessments between 2017 and 2024. Participants were retrospectively identified from four pre-existing clinical databases corresponding to the ASD, ADHD, SLD, and DMD groups. The study sample comprised a convenience clinical sample to obtain approximately balanced group sizes while including only participants with an available and valid CBCL/6–18 parent-report.

The sample size was not determined through an a priori power calculation. Instead, participants were selected from the available clinical databases according to the predefined inclusion criteria, aiming to obtain approximately balanced group sizes across the four diagnostic categories. Effect sizes were interpreted according to Cohen’s conventional benchmarks for η^2^ [28].

Diagnoses were established by board-certified child neuropsychiatrists according to DSM-5 criteria [1]. For ASD, standardized assessments included the Autism Diagnostic Observation Schedule—Second Edition (ADOS-2) [29] and the Autism Diagnostic Interview—Revised (ADI-R) [30]. ADHD diagnoses were supported by the Conners’ Parent and Teacher Rating Scales—Revised [31]. For SLD, standardized Italian measures of reading, writing, and mathematical abilities were administered, including the MT Reading Tests, AC-MT, and BVSCO [32,33,34,35,36,37,38]. DMD diagnoses were confirmed through neurological examination and molecular genetic testing identifying pathogenic variants in the dystrophin gene. Diagnostic procedures were performed within specialized clinical pathways to ensure consistency across evaluators. Although different clinicians were involved during the seven-year recruitment period as part of routine clinical practice, all participants were evaluated within the same tertiary pediatric hospital using standardized diagnostic procedures consistent with DSM-5 criteria and disorder-specific assessment protocols.

Inclusion criteria were: (a) confirmed diagnosis of one of the four target conditions; (b) age between 6 and 18 years; (c) availability of a completed CBCL/6–18 parent-report.

Exclusion criteria included: (a) comorbid diagnoses across the four target categories; (b) incomplete or invalid CBCL/6–18 data. Information on other comorbidities was not systematically collected and therefore could not be controlled for in the present analyses. This should be considered when interpreting the findings. No missing data were present in the final dataset.

### 2.2. Procedure

Data were retrospectively collected from clinical records and the corresponding diagnostic databases after completion of the routine clinical assessment. Only anonymized data were used for the present analyses.

The institutional ethics committee of the Catholic University of the Sacred Heart, Rome, Italy, approved the study (IRB number ID 5234), and all procedures performed in this study followed the standardized ethical guidelines of the institutional and national research committee and of the 1964 Helsinki Declaration and its later amendments, or comparable ethical standards. Informed consent to participate was obtained from the parents or legal guardians of all participants.

The study was reported in accordance with the Strengthening the Reporting of Observational Studies in Epidemiology (STROBE) guidelines [39], and the completed STROBE checklist is provided in the Supplementary Material.

### 2.3. Measures

#### 2.3.1. Demographic Information

Age and sex were collected from medical records.

#### 2.3.2. Assessment of Emotional and Behavioral Problems

Emotional and behavioral functioning was assessed using the CBCL/6–18, a standardized parent-report questionnaire from the Achenbach System of Empirically Based Assessment [4]. Parents rate the occurrence of 113 problem items on a 3-point scale (0 = not true, 1 = somewhat or sometimes true, 2 = very true or often true) based on the preceding 6 months. Raw scores are converted to age- and gender-normed T-scores.

The CBCL yields eight syndrome scales (Anxious/Depressed, Withdrawn/Depressed, Somatic Complaints, Social Problems, Thought Problems, Attention Problems, Rule-Breaking Behavior, Aggressive Behavior) and three broadband scales (Internalizing, Externalizing, and Total Problems). For syndrome scales, T-scores ≤ 64 are considered normal, 65–69 borderline, and ≥70 clinical. For broadband scales, ≤59 = normal, 60–64 = borderline, and ≥65 = clinical.

The CBCL/6–18 is widely used in pediatric psychology research and clinical practice to monitor emotional–behavioral functioning in children with chronic medical and developmental conditions, supporting both diagnosis-specific and transdiagnostic profiling.

### 2.4. Statistical Analysis

Descriptive statistics included means and standard deviations (SD) for continuous variables and frequencies (%) for categorical variables. The normality of continuous variables was assessed using the Shapiro–Wilk test. Preliminary analyses examined group differences in demographic characteristics using one-way analyses of variance (ANOVA) for continuous variables and chi-square (χ^2^) tests for categorical variables.

Group differences in CBCL/6–18 T-scores were primarily examined using one-way analyses of variance (ANOVA). The assumption of homogeneity of variances was assessed using Levene’s test. When this assumption was violated, Welch’s ANOVA was performed as a robust alternative. Following significant overall group effects, post hoc pairwise comparisons were conducted using Tukey’s Honest Significant Difference (HSD) test when homogeneity of variances was satisfied and Games–Howell tests when variances were unequal.

Effect sizes were estimated using eta squared (η^2^) and interpreted as small (η^2^ = 0.01), medium (η^2^ = 0.06), or large (η^2^ ≥ 0.14) according to Cohen [28]. *p* values less than or equal to 0.05 were considered statistically significant. All analyses were performed using IBM SPSS Statistics version 21.0 (IBM Corp., Armonk, NY, USA).

## 3. Results

### 3.1. Preliminary Statistical Analysis

All the variables met assumptions for parametric analyses, as indicated by the Shapiro–Wilk test (all *p*-values > 0.05).

### 3.2. Demographic Characteristics

The clinical sample included 197 children and adolescents: ASD (*n* = 47), ADHD (*n* = 50), SLD (*n* = 50), and DMD (*n* = 50).

The ASD group had a mean age of 8.7 years (SD = 3.82) and was 87% male (41/47); the ADHD group, 9.5 years (SD = 2.92), 90% male (45/50); the SLD group, 13.2 years (SD = 2.70), 58% male (29/50); and the DMD group, 11.5 years (SD = 2.59), 98% male (49/50).

Overall, males represented 83.2% of the sample. Group comparisons revealed significant differences in sex distribution, χ^2^(3) = 37.73, *p* < 0.001, with a higher proportion of females in the SLD group compared to the other groups. Significant age differences were also observed across diagnostic groups, F(3,193) = 16.31, *p* < 0.001. The SLD group showed the highest mean age among the four diagnostic groups. Detailed demographic data are presented in Table 1.

### 3.3. Emotional and Behavioral Problems

In the ASD group, mean CBCL/6–18 T-scores were 60.9 (SD = 11.8) for Internalizing, 56.2 (SD = 9.81) for Externalizing, and 60.7 (SD = 8.74) for Total Problems. Clinical-level scores were observed in 30% for Internalizing, 22% for Externalizing, and 30% for Total Problems. At the subscale level, Anxiety/Depression (I) showed the highest prevalence of clinical scores (13%), whereas most other subscales remained within the normal range.

In the ADHD group, mean T-scores were 67.0 (SD = 8.77) for Internalizing, 64.9 (SD = 7.19) for Externalizing, and 68.4 (SD = 6.64) for Total Problems. Rates of clinical-level scores were high (Internalizing = 76%, Externalizing = 60%, Total Problems = 76%), with peak elevations in Anxiety/Depression (I) (32%) and Attention Problems (VI) (24%).

In the SLD group, mean T-scores were 66.3 (SD = 10.7) for Internalizing, 55.5 (SD = 10.3) for Externalizing, and 61.4 (SD = 12.3) for Total Problems. Clinical scores were present in 66% for Internalizing, 18% for Externalizing, and 46% for Total Problems. Somatic Complaints (III) (54%) and Anxiety/Depression (I) (38%) emerged as the most affected subscales.

In the DMD group, mean T-scores were 55.7 (SD = 11.2) for Internalizing, 49.7 (SD = 9.69) for Externalizing, and 51.7 (SD = 11.3) for Total Problems. Clinical-level scores occurred in 26% for Internalizing, 4% for Externalizing, and 14% for Total Problems. Although most subscales fell within the normal range, Withdrawn/Depression (II) (14%) showed the highest rate of clinical scores, followed by Somatic Complaints (III) (8%) and Anxiety/Depression (I) (4%).

Overall, internalizing problems were more frequently observed than externalizing problems across the four diagnostic groups. Detailed descriptive statistics are reported in Table 2 and Table 3.

### 3.4. Comparative Analysis of Emotional and Behavioral Problems

**Figure 1:**
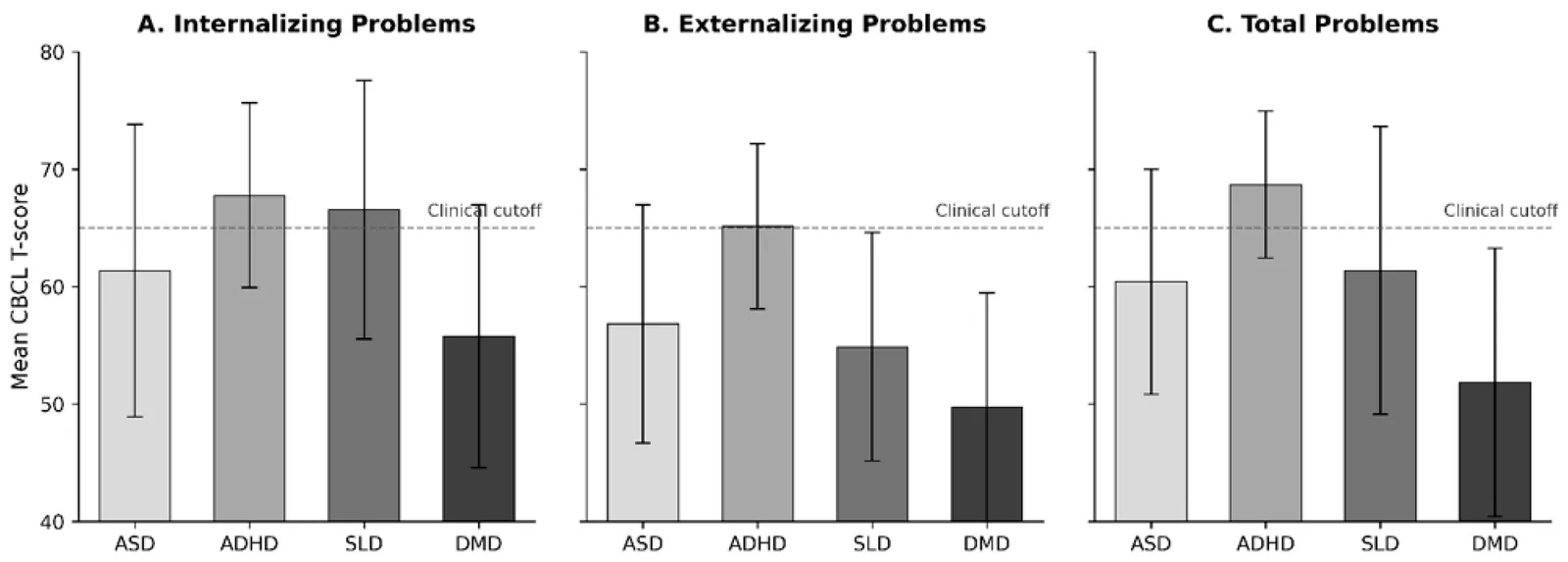
Mean CBCL/6–18 T-scores for Internalizing, Externalizing, and Total Problems across ASD, ADHD, SLD, and DMD groups. **Abbreviations:** ASD, Autism Spectrum Disorder; ADHD, Attention-Deficit/Hyperactivity Disorder; SLD, Specific Learning Disorders; DMD, Duchenne Muscular Dystrophy. Bars represent mean CBCL/6–18 T-scores and error bars indicate ±1 standard deviation (SD). The dashed horizontal line indicates the CBCL clinical cutoff (T = 65). Statistical comparisons are reported in Table 4.

Comparative analyses revealed significant differences in CBCL/6–18 Internalizing Problems, Externalizing Problems, and Total Problems across the ASD, ADHD, SLD, and DMD groups (Table 4; Figure 1).

For Internalizing Problems, Welch’s ANOVA revealed a significant main effect of diagnostic group (F(3, 104.84) = 14.26, *p* < 0.001, η^2^ = 0.168), indicating a large effect size. Post hoc Games–Howell comparisons showed that the ADHD group had significantly higher Internalizing scores than the ASD group (mean difference = 6.42, 95% CI [0.83, 12.01], *p* = 0.018) and the DMD group (mean difference = 12.00, 95% CI [6.94, 17.06], *p* < 0.001). In addition, the SLD group showed significantly higher Internalizing scores than the DMD group (mean difference = 10.76, 95% CI [4.96, 16.56], *p* < 0.001). No other pairwise comparisons reached statistical significance.

For Externalizing Problems, one-way ANOVA demonstrated a significant main effect of diagnostic group (F(3, 193) = 24.12, *p* < 0.001, η^2^ = 0.273), indicating a large effect size. Post hoc Tukey HSD comparisons indicated that the ADHD group showed significantly higher Externalizing scores than the ASD (mean difference = 8.31, 95% CI [3.45, 13.17], *p* < 0.001), SLD (mean difference = 10.24, 95% CI [5.46, 15.02], *p* < 0.001), and DMD groups (mean difference = 15.42, 95% CI [10.64, 20.20], *p* < 0.001). In addition, the ASD group showed significantly higher Externalizing scores than the DMD group (mean difference = 7.11, 95% CI [2.25, 11.97], *p* = 0.001). The SLD group showed significantly higher scores than the DMD group (mean difference = 5.18, 95% CI [0.40, 9.96], *p* = 0.028).

For Total Problems, Welch’s ANOVA showed a significant main effect of diagnostic group (F(3, 102.77) = 30.53, *p* < 0.001, η^2^ = 0.264), indicating a large effect size. Post hoc Games–Howell comparisons revealed that the ADHD group had significantly higher Total Problems scores than the ASD (mean difference = 8.27, 95% CI [3.93, 12.62], *p* < 0.001), SLD (mean difference = 7.32, 95% CI [2.20, 12.44], *p* = 0.002), and DMD groups (mean difference = 16.86, 95% CI [12.02, 21.70], *p* < 0.001). Furthermore, the ASD group showed significantly higher Total Problems scores than the DMD group (mean difference = 8.59, 95% CI [2.99, 14.18], *p* < 0.001), and the SLD group scored significantly higher than the DMD group (mean difference = 9.54, 95% CI [3.35, 15.73], *p* < 0.001).

## 4. Discussion

### 4.1. Comparative Profiles Across Neurodevelopmental and Neuromuscular Conditions

The present study provides insights into the emotional and behavioral profiles of children and adolescents with ASD, ADHD, SLD, and DMD using the CBCL/6–18 questionnaire. By comparing neurodevelopmental (ASD, ADHD, and SLD) and neuromuscular (DMD) conditions within a single investigation, this study addresses a gap in the literature, as previous research has largely examined these populations separately. Furthermore, using a common assessment instrument across all diagnostic groups enabled direct comparisons within a shared methodological framework, strengthening the present study’s transdiagnostic perspective. Beyond the novelty of including DMD within a transdiagnostic framework, this comparison may contribute to a better understanding of both shared and disorder-specific patterns of emotional and behavioral functioning across heterogeneous developmental conditions.

The present findings revealed significant differences across diagnostic groups in Internalizing Problems, Externalizing Problems, and Total Problems. Overall, the ADHD group exhibited the highest emotional and behavioral burden across the three broadband CBCL scales, whereas lower scores were observed in the DMD group. These findings should, however, be interpreted in light of the heterogeneity of clinical presentations and the reliance on a single parent-report measure.

Consistent with prior literature, significant differences were observed among the groups across the Internalizing Problems, Externalizing Problems, and Total Problems scales. Notably, the ADHD group exhibited the highest scores on all three broadband scales, particularly for Externalizing Problems, consistent with previous literature describing elevated externalizing behaviors in children with ADHD [13,14]. Similarly, the elevated Total Problems scores observed in the ADHD group support previous evidence highlighting the pervasive nature of emotional and behavioral difficulties in this population. At the same time, because CBCL items substantially overlap with behavioral manifestations typical of ADHD, these findings may partially reflect the instrument’s sensitivity to externalizing symptomatology, as also highlighted by previous research.

Both the ASD and SLD groups showed elevated Internalizing Problems, although no significant differences emerged between these groups. This finding is consistent with research by Ooi et al. [8] and Bauminger et al. [9], which identified social withdrawal and anxiety/depression as prominent challenges in individuals with ASD, as well as with previous evidence documenting elevated internalizing symptoms in children with SLD [11]. Within the SLD group, Somatic Complaints and Anxiety/Depression represented the syndrome scales with the highest proportion of clinical-range scores. In contrast, Externalizing Problems remained lower than those observed in the ADHD group, consistent with the findings reported by Cristofani et al. [18]. These findings further characterize the syndrome-scale profiles associated with each diagnostic group and may help identify domains that warrant particular attention during psychosocial assessment and intervention. However, because within-group variability (e.g., cognitive functioning, adaptive skills, or unmeasured comorbidities) was not systematically assessed, the present findings should be interpreted as reflecting broad group-level trends rather than homogeneous disorder-specific profiles.

In contrast, the DMD group generally showed lower CBCL broadband scores than the ADHD group and, for Externalizing Problems and Total Problems, also lower scores than the ASD group. Compared with the SLD group, DMD showed a broadly similar profile for Internalizing Problems but lower scores for Externalizing and Total Problems. These findings suggest that neuromuscular disorders such as DMD may present a distinct emotional and behavioral profile compared with neurodevelopmental conditions. These lower rates of clinical-level problems in the DMD group should be interpreted with caution, as they may, in part, reflect the perspective of parents completing the CBCL/6–18. However, prior literature [19] has already raised concerns about the accuracy of parent-reported assessments, suggesting that certain difficulties—particularly internalizing symptoms—might be underestimated in clinical contexts. The present findings also indicated specific difficulties in Internalizing Problems, particularly in the Withdrawn/Depression and Somatic Complaints subscales. Hendriksen and Vles [23], Ricotti et al. [25], and Colombo et al. [26] previously noted that children with DMD often exhibit internalizing symptoms such as anxiety and depression. Still, the reliance on parental perspectives might underrepresent the full extent of these difficulties—especially given that caregivers face overwhelming responsibilities in managing a chronic and progressive condition. Moreover, the lower Externalizing Problems observed in DMD may partly reflect disease-related motor limitations, which could reduce the expression or detectability of overt disruptive behaviors.

Taken together, these findings highlight the value of multidimensional assessment approaches that capture both shared and disorder-specific patterns of functioning. More broadly, they support the clinical utility of a transdiagnostic perspective for identifying both common and disorder-specific emotional–behavioral characteristics across heterogeneous developmental conditions. Such approaches may contribute to more individualized clinical formulations and facilitate the interpretation of emotional–behavioral symptoms across heterogeneous developmental populations.

The present findings may be further interpreted within broader developmental frameworks that emphasize the interaction among biological vulnerabilities, individual psychological processes, and contextual influences across development [40,41]. Such perspectives may help contextualize variability in symptom expression across heterogeneous clinical populations.

Overall, these findings reinforce the need for transdiagnostic assessment models that integrate disorder-specific characteristics with broader developmental and contextual processes when evaluating emotional and behavioral functioning in children and adolescents.

### 4.2. Clinical Implications for Screening and Assessment

Although the present study was not designed to investigate neurobiological mechanisms directly, the previous literature has proposed several neurobiological models that may help contextualize emotional and behavioral manifestations across neurodevelopmental and neuromuscular conditions, including alterations involving dystrophin-related pathways, limbic circuitry, stress-response systems, and frontostriatal networks [42,43,44,45]. These mechanisms were not examined in the present study and are presented only as theoretical frameworks that may assist in interpreting the observed findings.

From a clinical perspective, the distinct emotional and behavioral profiles observed across diagnostic groups may have implications for developmentally informed screening and assessment strategies. Importantly, the following implications should be interpreted as hypothesis-generating clinical considerations rather than recommendations directly derived from the present data. Integrating behavioral observations with standardized parent–teacher reports may facilitate the early identification of at-risk profiles and support timely psychosocial interventions tailored to the child’s and family’s needs.

- Children with ADHD may particularly benefit from structured behavioral observations and multi-informant assessments to facilitate the early identification of disruptive behaviors and support timely behavioral and parent-focused interventions [46].
- For ASD, routine assessment may benefit from systematic screening for anxiety and mood symptoms alongside interventions targeting emotion regulation, adaptive functioning, and social competence [47,48].
- For SLD, considering somatic symptoms within psychoeducational evaluations may help identify children experiencing substantial emotional distress and guide interventions aimed at strengthening coping skills and resilience [49].
- For DMD, longitudinal monitoring combining standardized measures with caregiver interviews may facilitate the identification of emotional difficulties that could otherwise remain overshadowed by the physical manifestations of the disease, thereby supporting comprehensive multidisciplinary care.

Taken together, these considerations illustrate how a transdiagnostic assessment framework may facilitate individualized clinical decision-making by integrating behavioral, developmental, and contextual information across heterogeneous pediatric populations.

### 4.3. Comparison with Previous Literature and Potential Contextual Factors

While our findings align with many established patterns, several discrepancies with prior research warrant attention. For example, the relatively lower levels of reported emotional and behavioral difficulties in the DMD group appear to differ from earlier studies reporting higher rates of anxiety, depression, and social withdrawal in this population [23,50]. These differences may reflect methodological variations—such as reliance on parental CBCL reports versus multi-informant or clinician-rated assessments—or contextual factors including caregiver adaptation to chronic illness. Variability in sample composition and clinical characteristics across studies may have further contributed to these divergent findings.

In ASD, lower Externalizing scores than those reported by Totsika et al. [51] may be related to differences in sample characteristics, assessment procedures, or variability in symptom presentation across ASD populations. In ADHD, the higher Total Problems scores observed here—relative to some community-based studies [52]—may reflect the clinical nature of the present sample, which likely differs from community-based cohorts in symptom severity and referral characteristics. For SLD, discrepancies with Klassen and Lynch [53] may indicate that contextual and educational factors could contribute to emotional adjustment, although such variables were not assessed in the present study.

From a contextual perspective, these divergences may reflect the potential influence of healthcare system organization, family coping styles, and cultural attitudes toward disability. Although these contextual factors were not directly assessed in the present study, they represent plausible explanations that warrant further investigation. In environments where specialized psychosocial services are scarce, parents may differ in how they perceive and report emotional or behavioral difficulties, particularly in chronic physical conditions where medical management dominates daily routines. Such variability suggests the potential value of screening approaches that are sensitive to contextual differences and can identify at-risk children even in under-resourced settings.

These observations are also consistent with ecological developmental models emphasizing the role of multiple environmental systems—from family and school contexts to broader sociocultural influences—in shaping emotional and behavioral functioning [54].

At the same time, because these contextual influences were not directly measured, their contribution to the observed differences should be interpreted cautiously. This consideration also highlights several methodological limitations that should be taken into account when interpreting the present findings.

### 4.4. Strengths, Limitations, and Future Directions

The present study has several strengths that should be considered when interpreting its findings. First, it adopted a transdiagnostic design that enabled the direct comparison of emotional and behavioral profiles across children and adolescents with ASD, ADHD, SLD, and DMD using the same standardized assessment instrument (CBCL/6–18). This approach minimized methodological variability associated with differences in assessment procedures and facilitated the identification of both shared and disorder-specific patterns across diagnostically heterogeneous clinical populations. Second, the inclusion of a naturalistic clinical sample enhances the ecological validity of the findings by reflecting routine clinical practice. Finally, the inclusion of children with DMD, a population that remains underrepresented in comparative studies of emotional and behavioral functioning, broadens the current evidence base and contributes to extending transdiagnostic research beyond neurodevelopmental disorders alone.

Despite these strengths, several limitations should be considered when interpreting the present findings. First, the study relied exclusively on parent-reported CBCL/6–18 data. Although the CBCL is a widely validated instrument [39], parent reports may be influenced by caregiver perceptions, stress levels, and cultural norms, potentially leading to under- or over-reporting of specific emotional and behavioral symptoms. Furthermore, the interpretability of CBCL profiles may vary across diagnostic groups, particularly in heterogeneous clinical populations such as ASD and DMD, where atypical developmental trajectories, communicative characteristics, or disease-related features may influence both symptom expression and parental recognition of emotional difficulties [6,7,19,24]. Consequently, the exclusive use of parent-report measures may not fully capture the complexity of emotional and behavioral functioning across these populations, supporting the importance of complementing caregiver questionnaires with multi-informant assessments and direct clinical observations [15,16,19].

Several additional methodological limitations should also be acknowledged. The relatively small sample size, particularly for DMD given the condition’s rarity, may limit the generalizability of the findings. However, the inclusion of all eligible patients assessed during the study period allowed the study to reflect a naturalistic clinical population. This approach enhances the ecological validity of the findings, especially for underrepresented conditions such as DMD, which remain rarely represented in comparative behavioral research.

Furthermore, the absence of an a priori sample size calculation may have limited the statistical power to detect smaller between-group differences and should therefore be considered when interpreting the results. An additional limitation is that some syndrome-scale frequencies were based on small numbers of participants and should therefore be interpreted with caution.

In addition, information regarding cognitive functioning, symptom severity, medication use, socioeconomic characteristics, parental educational level, treatment history, and other clinical variables was not systematically available across all diagnostic groups. It therefore could not be included in the analyses. Because these factors may influence both emotional–behavioral functioning and parental reporting patterns, their potential contribution to between-group differences cannot be excluded. It should be considered when interpreting the present findings.

Finally, the predominance of male participants across diagnostic groups reflects the epidemiology of many neurodevelopmental and neuromuscular disorders but may nevertheless limit the generalizability of the findings to female populations. In addition, the unequal distribution of age and sex across the diagnostic groups may have partially contributed to the observed between-group differences. Although the outcome measures were based on CBCL/6–18 T-scores standardized for age and sex, residual confounding related to demographic differences cannot be completely excluded and should therefore be considered when interpreting the findings. Moreover, the cross-sectional design precludes conclusions regarding developmental trajectories or causal relationships.

Future research should extend the present findings through longitudinal designs and multi-informant assessment strategies integrating parent reports with teacher questionnaires, clinician-administered measures, and objective behavioral assessments [55]. Such approaches would provide a more comprehensive characterization of emotional and behavioral functioning and help clarify developmental trajectories across neurodevelopmental and neuromuscular conditions. In addition, applying multivariate statistical approaches may provide a more integrated characterization of the overall behavioral profile across diagnostic groups by simultaneously examining correlated emotional and behavioral domains.

Future investigations should also examine the influence of cognitive functioning, symptom severity, treatment history, medication use, socioeconomic characteristics, parental educational level, and other contextual factors on emotional and behavioral outcomes. In addition, further transdiagnostic investigations are needed to evaluate the feasibility and clinical utility of integrated assessment and care models in which psychological support is embedded within routine pediatric follow-up.

Such approaches may help improve early identification, individualized care planning, adaptive functioning, and quality of life across heterogeneous developmental populations [56], ultimately supporting the development of more personalized and developmentally informed models of psychological assessment and care.

## 5. Conclusions

In conclusion, the present study contributes to the understanding of emotional and behavioral functioning across neurodevelopmental and neuromuscular conditions by providing a transdiagnostic comparison of children and adolescents with ASD, ADHD, SLD, and DMD using a common standardized assessment instrument (CBCL/6–18). This approach enabled the identification of both shared and disorder-specific emotional and behavioral patterns across diagnostically heterogeneous clinical populations. The findings reveal important group-specific differences, such as greater externalizing difficulties in ADHD, more pronounced internalizing features in ASD, distinct emotional–behavioral characteristics in SLD, and relatively lower parent-reported emotional and behavioral difficulties in the DMD group. Importantly, these findings should be interpreted with caution, particularly for DMD, as lower reported difficulties may reflect differences in symptom expression or limitations inherent to parent-report measures rather than a reduced psychological burden.

From a clinical perspective, the present findings support the value of multidimensional, multi-informant assessment strategies that capture both shared and disorder-specific emotional and behavioral characteristics across heterogeneous developmental conditions. Embedding emotional and behavioral screening within broader biopsychosocial and developmental frameworks may facilitate earlier identification of emerging difficulties and contribute to more individualized assessment and care planning.

Future research should adopt longitudinal designs integrating multi-informant assessments, objective behavioral measures, and broader clinical and contextual variables to better characterize developmental trajectories and emotional–behavioral profiles across neurodevelopmental and neuromuscular conditions. Such efforts may contribute to more sensitive assessment and screening practices, ultimately supporting more individualized and developmentally informed models of psychological assessment and care for these populations.

## Figures and Tables

**Table 1:** Demographic Characteristics of the Sample.

Variables	Total Sample (*n* = 197) *n* (%) or Mean (SD) *	ASD (*n* = 47) *n* (%) or Mean (SD) *	ADHD (*n* = 50) *n* (%) or Mean (SD) *	SLD (*n* = 50) *n* (%) or Mean (SD) *	DMD (*n* = 50) *n* (%) or Mean (SD) *	Statistic F or χ^2^°	*p*
Age, Years *	10.8 (3.03)	8.7 (3.82)	9.5 (2.92)	13.2 (2.70)	11.5 (2.59)	16.31	**<0.001**
Sex, *n* (%)°							
*Male*	164 (83.2)	41 (87)	45 (90)	29 (58)	49 (98)	37.73	**<0.001**
*Female*	33 (16.8)	6 (13)	5 (10)	21 (42)	1 (2)		

**Abbreviations:** N, number; SD, standard deviation; F, F statistic from one-way ANOVA; χ^2^, chi-square statistic; ASD, Autism Spectrum Disorder; ADHD, Attention-Deficit/Hyperactivity Disorder; SLD, Specific Learning Disorders; DMD, Duchenne Muscular Dystrophy. Age was compared using one-way ANOVA and sex using the χ^2^ test. Values in bold indicate statistically significant scores.* Values are expressed as mean (SD).

**Table 2:** Descriptive Statistics and Clinical Classification of CBCL/6–18 Main Scales.

Variables	ASD (*n* = 47) *n* (%) or Mean (SD) *	ADHD (*n* = 50) *n* (%) or Mean (SD) *	SLD (*n* = 50) *n* (%) or Mean (SD) *	DMD (*n* = 50) *n* (%) or Mean (SD) *
Internalizing *	60.9 (11.8)	67.0 (8.77)	66.3 (10.7)	55.7 (11.2)
*Clinical Level*	14 (30)	38 (76)	33 (66)	13 (26)
*Borderline Level*	12 (26)	5 (10)	2 (4)	8 (16)
Externalizing *	56.2 (9.81)	64.9 (7.19)	55.5 (10.3)	49.7 (9.69)
*Clinical Level*	10 (22)	30 (60)	9 (18)	2 (4)
*Borderline Level*	12 (27)	8 (16)	8 (16)	7 (14)
Total Problems *	60.7 (8.74)	68.4 (6.64)	61.4 (12.3)	51.7 (11.3)
*Clinical Level*	14 (30)	38 (76)	23 (46)	7 (14)
*Borderline Level*	12 (27)	5 (10)	9 (18)	5 (10)

Continuous variables are reported as mean T-scores (SD). Clinical and Borderline Levels indicate the number and percentage of participants who meet the CBCL standardized cutoffs. * Values are expressed as mean (SD).

**Table 3:** Frequencies of Clinical and Borderline Scores Across CBCL/6–18 Syndrome Subscales.

Subscales	ASD (*n* = 47) *n* (%)	ADHD(*n* = 50) *n* (%)	SLD (*n* = 50) *n* (%)	DMD (*n* = 50) *n* (%)
**Anxiety/Depression (I)**				
*Clinical Level*	6 (13)	16 (32)	19 (38)	2 (4)
*Borderline Level*	2 (4)	17 (34)	6 (12)	6 (12)
**Withdrawn/Depression (II)**				
*Clinical Level*	2 (4)	4 (8)	14 (28)	7 (14)
*Borderline Level*	3 (6)	8 (16)	8 (16)	2 (4)
**Somatic Complaints (III)**				
*Clinical Level*	2 (4)	10 (20)	27 (54)	4 (8)
*Borderline Level*	2 (4)	6 (12)	7 (14)	3 (6)
**Social Problems (IV)**				
*Clinical Level*	1 (2)	7 (14)	7 (14)	0 (0)
*Borderline Level*	2 (4)	12 (24)	12 (24)	6 (12)
**Thought Problems (V)**				
*Clinical Level*	1 (2)	8 (16)	12 (24)	1 (2)
*Borderline Level*	1 (4)	7 (14)	5 (10)	3 (6)
**Attention Problems (VI)**				
*Clinical Level*	1 (2)	12 (24)	9 (18)	1 (2)
*Borderline Level*	2 (4)	10 (20)	9 (18)	2 (4)
**Rule-Breaking Behavior (VII)**				
*Clinical Level*	1 (2)	5 (10)	3 (6)	0 (0)
*Borderline Level*	0 (0)	3 (6)	3 (6)	1 (2)
**Aggressive Behavior (VIII)**				
*Clinical Level*	0 (0)	5 (10)	5 (10)	1 (2)
*Borderline Level*	1 (2)	5 (10)	7 (14)	2 (4)

Clinical and Borderline Levels indicate the number and percentage of participants exceeding CBCL standardized thresholds. Syndrome subscale frequencies are presented descriptively and were not subjected to inferential statistical comparisons.

**Table 4:** One-way ANOVA/Welch ANOVA and Post Hoc Comparisons for CBCL/6–18 Main Scales.

Variables	Test	F-Value (df1, df2)	*p*-Value	η^2^	ADHD vs. ASD	ASD vs. SLD	ASD vs. DMD	ADHD vs. SLD	ADHD vs. DMD	SLD vs. DMD
**Internalizing**	Welch ANOVA	14.26 (3, 104.84)	**<0.001**	0.168						
*p-value*					**0.018**	0.141	0.102	0.916	**<0.001**	**<0.001**
**Externalizing**	One-way ANOVA	24.12 (3, 193)	**<0.001**	0.273						
*p-value*					**<0.001**	0.732	**0.001**	**<0.001**	**<0.001**	**0.028**
**Total Problems**	Welch ANOVA	30.53 (3, 102.77)	**<0.001**	0.264						
*p-value*					**<0.001**	0.973	**<0.001**	**0.002**	**<0.001**	**<0.001**

**Abbreviations:** ANOVA, analysis of variance; Welch ANOVA, Welch analysis of variance; df, degrees of freedom; η^2^, eta squared. Group comparisons were performed using one-way ANOVA or Welch ANOVA depending on the results of Levene’s test for homogeneity of variances. Post hoc pairwise comparisons were conducted using Tukey’s Honest Significant Difference (HSD) test when homogeneity of variances was satisfied and Games–Howell tests when variances were unequal. η^2^ values represent effect sizes. Values in bold indicate statistically significant pairwise comparisons. The direction of significant pairwise differences is indicated by reporting first the group with the higher mean CBCL T-score.

## Data Availability

The data presented in this study are available on request from the corresponding author. The data are not publicly available due to privacy and ethical restrictions.

## Supplementary Materials

The following supporting information can be downloaded at: https://www.mdpi.com/article/10.3390/children13080996/s1, STROBE Checklist.
