# Supplementary material for: Emotional and Behavioral Profiles in Neurodevelopmental and Neuromuscular Disorders: A Comparative Study Using the Child Behavior Checklist
Source: Children (Basel). 2026 Jul 27;13(8):996. doi: 10.3390/children13080996 (PMC13511199; doi:10.3390/children13080996)
Supplement: Supplementary file 1 [file children-13-00996-s001.zip › children-4382478-supplementary.pdf]

STROBE Statement—Checklist of items that should be included in reports of ***cross-sectional studies***

	Item No	Recommendation	Reported	Location
**Title and abstract**	1	(*a*) Indicate the study's design with a commonly used term in the title or the abstract	Yes	Abstract: Methods
		(*b*) Provide in the abstract an informative and balanced summary of what was done and what was found	Yes	Abstract (entire section)
**Introduction**				
Background/rationale	2	Explain the scientific background and rationale for the investigation being reported	Yes	Introduction (paragraphs 1-12)
Objectives	3	State specific objectives, including any prespecified hypotheses	Yes	Introduction (final paragraph)
**Methods**				
Study design	4	Present key elements of study design early in the paper	Yes	Methods (2.1 Participants)
Setting	5	Describe the setting, locations, and relevant dates, including periods of recruitment, exposure, follow-up, and data collection	Yes	Methods (2.1 Participants; 2.2 Procedure)
Participants	6	(*a*) Give the eligibility criteria, and the sources and methods of selection of participants	Yes	Methods (2.1 Participants)
Variables	7	Clearly define all outcomes, exposures, predictors, potential confounders, and effect modifiers. Give diagnostic criteria, if applicable	Yes	Methods (2.3 Measures; 2.4 Statistical Analysis)
Data sources/ measurement	8*	For each variable of interest, give sources of data and details of methods of assessment (measurement). Describe comparability of assessment methods if there is more than one group	Yes	Methods (2.3 Measures)
Bias	9	Describe any efforts to address potential sources of bias	Yes	Discussion (4.4 Strengths, Limitations, and Future Directions)
Study size	10	Explain how the study size was arrived at	Yes	Methods (2.1 Study Design); Discussion (4.4 Strengths, Limitations, and Future Directions)
Quantitative variables	11	Explain how quantitative variables were handled in the analyses. If applicable, describe which groupings were chosen and why	Yes	Methods (2.4 Statistical Analysis)
Statistical methods	12	(*a*) Describe all statistical methods, including those used to control for confounding	Yes	Methods (2.4 Statistical Analysis)
		(*b*) Describe any methods used to examine subgroups and interactions	N.A.	Not applicable (no subgroup or interaction analyses were performed)
		(*c*) Explain how missing data were addressed	Yes	Methods (2.1 Participants)

		(*d*) If applicable, describe analytical methods taking account of sampling strategy	N.A.	Not applicable (sampling strategy not applicable)
		(*e*) Describe any sensitivity analyses	N.A.	Not applicable (no sensitivity analyses were performed)
**Results**				
Participants	13*	(a) Report numbers of individuals at each stage of study—eg numbers potentially eligible, examined for eligibility, confirmed eligible, included in the study, completing follow-up, and analysed	Yes	Methods (2.1 Participants); Results (3.2 Demographic Characteristics)
		(b) Give reasons for non-participation at each stage	Yes	Methods (2.1 Participants)
		(c) Consider use of a flow diagram	N.A.	Not applicable (no participant flow diagram included)
Descriptive data	14*	(a) Give characteristics of study participants (eg demographic, clinical, social) and information on exposures and potential confounders	Yes	Results (3.2 Demographic Characteristics), Table 1
		(b) Indicate number of participants with missing data for each variable of interest	Yes	Methods (2.1 Participants)
Outcome data	15*	Report numbers of outcome events or summary measures	Yes	Results (3.3 Emotional and Behavioral Problems Assessments; 3.4 Comparative Analysis of Emotional and Behavioral Problems); Tables 2–4; Figure 1
Main results	16	(*a*) Give unadjusted estimates and, if applicable, confounder-adjusted estimates and their precision (eg, 95% confidence interval). Make clear which confounders were adjusted for and why they were included	Yes	Results (3.4 Comparative Analysis of Emotional and Behavioral Problems)
		(*b*) Report category boundaries when continuous variables were categorized	Yes	Methods (2.3 Measures)
		(*c*) If relevant, consider translating estimates of relative risk into absolute risk for a meaningful time period	N.A.	Not applicable
Other analyses	17	Report other analyses done—eg analyses of subgroups and interactions, and sensitivity analyses	N.A.	Not applicable (no subgroup, interaction, or sensitivity analyses were performed)
**Discussion**				
Key results	18	Summarise key results with reference to study objectives	Yes	Discussion (4.1 Comparative Profiles Across

				Neurodevelopmental and Neuromuscular Conditions)
Limitations	19	Discuss limitations of the study, taking into account sources of potential bias or imprecision. Discuss both direction and magnitude of any potential bias	Yes	Discussion (4.4 Strengths, Limitations, and Future Directions)
Interpretation	20	Give a cautious overall interpretation of results considering objectives, limitations, multiplicity of analyses, results from similar studies, and other relevant evidence	Yes	Discussion (4.1 Comparative Profiles Across Neurodevelopmental and Neuromuscular Conditions; 4.2 Clinical Implications for Screening and Assessment; 4.3 Comparison with Previous Literature and Potential Contextual Factors; 4.4 Strengths, Limitations, and Future Directions; Conclusions)
Generalisability	21	Discuss the generalisability (external validity) of the study results	Yes	Discussion (4.4 Strengths, Limitations, and Future Directions)
**Other information**				
Funding	22	Give the source of funding and the role of the funders for the present study and, if applicable, for the original study on which the present article is based	Yes	Funding

*Give information separately for exposed and unexposed groups.

Note: An Explanation and Elaboration article discusses each checklist item and gives methodological background and published examples of transparent reporting. The STROBE checklist is best used in conjunction with this article (freely available on the Web sites of PLoS Medicine at http://www.plosmedicine.org/, Annals of Internal Medicine at http://www.annals.org/, and Epidemiology at http://www.epidem.com/). Information on the STROBE Initiative is available at www.strobe-statement.org.
